# Efficacy of non -thermal pressure plasma versus other modalities for disinfection of primary root canals

**DOI:** 10.1186/s12903-024-05349-5

**Published:** 2025-01-11

**Authors:** Shaymaa A. El Shishiny, Yomna O. Morad, Rania I. Hindi, Amina M. El-Motasem, Asmaa A. El Sheshiny, Dalia M. Alramady, Amira M. Samy

**Affiliations:** 1 Lecturer of Pedodontics and Oral Health, Faculty of Dental Medicine for Girls, Al Azhar University, Cairo, Egypt; 2 Health Radiation Research Department, National Center for Radiation Research and Technology, Egyptian Atomic Energy Authority, Cairo, Egypt; 3 Lecturer of Biophysics, Physics Department, Faculty of Science for Girls, Al-Azhar University, Cairo, Egypt; 4 Lecture of Operative Dentistry, Luminous Technical University College, Medical and Applied Department, Dental Technology, Amman, Jordan; 5 Associate Professor of Operative Dentistry, Conservative Dentistry Department, Faculty of Oral and Dental Medicine Badr University in Cairo, Cairo, Egypt

**Keywords:** Primary teeth, Root canal therapy, Root canal irrigant, Enterococcus Fecalis, NTPP, Diode laser, Propolis, CHX

## Abstract

**Background:**

Endodontic treatment aims in the preservation of extremely carious primary teeth. For root canal therapy to be successful, root canals must be properly prepared and effectively irrigated .Therefore, it is necessary to select the proper root canal disinfection method to preserve the primary tooth.

**Objective:**

This research was carried out to compare non-thermal pressure plasma (NTPP), diode laser, propolis, and chlorhexidine (CHX) efficacy for disinfection of deciduous anterior root canals contaminated with Enterococcus Faecalis (E. faecalis) after sterilization by gamma radiation.

**Methods:**

In this study, forty extracted single-rooted primary anterior teeth were used. All teeth were cleaned, disinfected, and stored till use. Gaining access was provided till reaching the orifices of canals, all pulp tissue debris was removed, and root canals of all teeth were prepared. Standardized 8 mm root length was obtained through crown decronation below the cemento-enamel junction. Samples were sterilized by gamma radiation then the bacterial suspension was inoculated inside root canals. Specimens divided into four main groups; ten samples each group. Group I: Samples irrigated with chlorhexidine. Group II: Samples treated with diode laser. Group III: Samples irrigated with Ethanolic extract of propolis. Group IV: Samples treated with NTPP.

**Results:**

A significant difference was found between values measured before and after four irrigation types (*p* < 0.001) for CHX, Diode Laser, NTPP and (*P* = 0.035) for Propolis. The highest values of colony reduction measured before and after irrigation were for NTPP (4.06 ± 0.88**)**. Maximum reduction in colony-forming units was recorded in the NTPP group (98.79%), while the lowest reduction in colony-forming units was recorded in Propolis group (81.99%).

**Conclusion:**

All tested methods (CHX, NTPP, Diode laser and Propolis) decreased colony count, with the highest reduction noted in group treated by NTPP and the least reduction noticed in Propolis treated group.

**Supplementary Information:**

The online version contains supplementary material available at 10.1186/s12903-024-05349-5.

## Introduction

Early exfoliation of primary teeth causes esthetic, biological, and functional problems [1]. Thus the goal of treatment of deciduous teeth with large carious lesions is dental arch preservation, restoring healthy condition of the affected pulpal tissues, and maintaining normal development of permanent successors. Pulpectomy eliminates microorganisms from root canals by chemical irrigation and mechanical debridement [2]. Its success depends on optimum root canal irrigation and effective disinfection. Enterococcus faecalis is considered a survivor of the chemo-mechanical steps, with its virulence factors that promote its adhesion to host cells. It can resist nutrient deprivation in endodontically treated teeth and attach to the collagen present in the dentin, showing resistance to chemo-mechanical procedures [3]. Root canal is an enclosed complex anatomical space with morphological and microbiological challenges. Usual instrumentation techniques pile debris in the isthmus areas so; the ideal irrigant should be a biocompatible bactericidal, pulp tissue solvent, lubricating agent, smear layer and debris remover, and with sustained effect but without influencing dentin physical properties [4, 5]. 

Several adopted modes for root canal sanitization in deciduous teeth are found in the literature. Sodium hypochlorite (NaOCl) is the gold standard irrigant by virtue of its apparent antibacterial characteristics, ease of manipulation, decreased surface tension, and cheapness. However, it can negatively affect the permanent successors’ dental follicles in the presence of root resorption [4]. Chlorhexidine is a sustained broad-spectrum antimicrobial irrigating solution with low toxicity. CHX is a synthetic cationic biguanide that interacts with the negative charges of the phosphate groups on the microbial wall, resulting in osmotic un-equilibrium of the cell [6]. 

A diode laser has various applications in dentistry with promising disinfection outcomes [7]. 

The bactericidal explicit impact of a diode laser (810 nm, penetrates more than 1 mm through dentinal thickness) depends on thermal affection; bacteria cannot resist laser exposure [8, 9]. 

Plasma medicine has drawn increased attention in biomedical fields including sterilization, surface modification, and cancer treatments. Plasma is the fourth state of matter, where atmosphere pressure nonequilibrium plasma (APNP) jet generates active radicles and charged particles that have antimicrobial activity in open space at room temperature [10]. NTPP jet has been applied in various dental studies to effectively deactivate major pathogenic microorganisms in oral cavity and their biofilms such as Pseudomonas aeruinosa, Streptcoccus mutans, and enterococcus faecalis [11]. Moreover the antimicrobial characteristics, NTPPs have shown anti-inflammatory trails, antifungal, antiparasitic, antiviral properties, and tissue repair [12, 13]. Also, it was reported that NTPP as a prosperous tool used as an adjunctive therapy against malignancy [14, 15].

NTPPs have two modes of action and both are efficient in dentistry; direct action by applying NTPP plasma plume to a set substrate, or circuitous action, through liquid activation by NTPP before its application to the substrate [16, 17]. 

Propolis is a wax-like biomaterial gained naturally from beehives; it has a wide range of biomedical implementations such as tissue regeneration, ulcers, oral infections, skin lesions, herpes, and wound healing [18]. 

Various endodontic usage of propolis through intracanal irrigation [19, 20] intracanal medication (21, 22), post-endodontic pain relief [23, 24] and vital pulp therapy have been examined [25]. 

Therefore, this study compares the efficacy of NTPP, diode laser, propolis, and CHX for disinfection of deciduous anterior root canals infected with E. faecalis after sterilization by gamma radiation.

## Methods

### Teeth selection

Forty primary anterior teeth were collected from faculty of dental medicine for girls’ Al-Azhar University outpatient dental clinics after obtaining a written consent from the accompanying parent for using the teeth for research purposes; [26–28] research guidelines of ethics committee with approval code (REC-PD-24-03) were followed.

### Regulatory statement

No human or living tissue participation in this study, so it was exempt from clinical trial registration.

All teeth were picked according to the next criteria: [29]

### A-Inclusion criteria

- Primary anterior teeth.
- Free from internal or external resorption.
- Complete root formation.

### Mechanical preparation of teeth

Teeth cleansing was done to remove any tissue debris using an ultrasonic scaler, rinsed and disinfected with 1% Naocl, and then stored in thymol preservative till use. Access cavity was prepared using a round bur and tapered diamond bur (Jiangsu-China) till reaching the orifices of canals, pulp tissue was extirpated using H-file (Mani Japan) then root canal preparation was completed by Kido-s rotary files (India). (30،^31^)

Organic pulp tissue remnants were rinsed with 1 ml of 1% Naocl while the inorganic smear layer of root dentin was removed using 17% EDTA with each file; the canals were then flushed with 0.9% saline to remove residual irrigants and stop their effects [5]. 

### Teeth decoronation

Teeth coronectomy below the cementoenamel junction was done till having a standardized 8 mm root length [32] using a table motor with diamond size under coolant water spray. These root canals with standard lengths act as reservoirs for bacterial suspension [32]. Light-cured composite resin was used to seal the apical foramen to prevent the extrusion of bacterial suspension and irrigant solutions.

### Samples sterilization

Root samples were inserted into sterilization pouches after mechanical preparation and sterilized by gamma irradiation (Cobalt 60 irradiator of 1.774 KGY dose rate, total dose of 25 KGY, Egyptian Atomic Energy Authority).

### Sterility test

Before injection of bacterial suspension, a sterility test was done to ensure bacterial (gram positive or gram negative) and fungal decontamination of the sterilized root samples. These were done by injection of the root canals with sterile saline, and then insertion of three sterile absorbent paper points into the root canals for one minute until they became saturated with the injected fluid and then transferred into:.

- Nutrient Agar (to exclude gram + ve bacterial contamination).
- MacConkey’s agar (to exclude gram -ve bacterial contamination).
- Sabouraud dextrose agar (to exclude fungal contamination).

Plates incubation at 37° for 24 h under observation was done, to check effective sterilization if they remain clear.

### Sample size calculation

Sample size has been determined to be ten samples per group referenced to [33] feigning α = 0.05, β = 0.2, mean standard deviation of log colony-forming units to be 2.9 and practical size of 0.76 utilizing one-way analysis of variance (ANOVA) by G*Power 3.1.9.4 software.

### Specimens grouping

They were divided into four groups; each group consisted of ten samples:

Group I: irrigated with chlorhexidine.

Group II: treated with diode laser (810 mm).

Group III: irrigated with ethanolic extract of propolis.

Group IV: treated with NTPP.

.

### Propolis extracts preparation

Extraction of propolis was carried out by maceration with 96% ethanol. Three hundred grams of propolis were measured by digital electronic scale and were combined with 300 ml 96% ethanol at 37^o^C to obtain 100% (w\v) extract, then stored in a bottle closed tightly for one week. Then, the supernatant was filtered using a chromafil filter to eliminate impurities [34]. 

### Selection and preparation of bacterial microorganisms

#### I-Bacterial reference strain

Enterococcus faecalis references strain American Type of Culture Collection (ATCC 49533) was kindly provided by the regional center of mycology and biotechnology Al Azhar University of Egypt, to be used in this study, it was supplied as an actively growing culture on slope agar.

#### II- Bacterial substructure preparation

Microorganisms were preserved at -70℃ in brain heart infusion broth with 15% glycerol, freshly set subculture on bile auscline medium and previously incubated for 24 h at 37⁰ C.

#### III- Inoculation of bacteria

Forcible injections of 1 ml of bacterial suspension were carried out to be sure of reaching the entire working length of each root canal by a sterile syringe; samples then were placed individually submerged with 2 ml of brain heart infusion broth inside tightly sealed Eppendorf tubes (Fisherscl. Co.Uk) then incubated at 37℃ for 24 h for allowing bacterial multiplication [35]. 

#### IV- Bacterial count

First microbial sample (S1) was driven from each root canal by inserting three successive sizes [30, 35, 40] of sterile absorbent paper points (Diadent- Co, Korea.) into each root canal for one minute till being fully satiated with bacteria [32], then removed and added to1ml saline in a sterile falcon tube.

A sequent 10-fold dilution of microbial suspension in sterile saline (1/10, 1/100, 1/1000, 1/10000, 1/100000) was prepared using a micropipette, 0.1 ml from each dilution was plated on the brain heart infusion agar plates using the bacteriologic loop then incubated at 37°Cfor 24 h.

Colony count through multiplying the amount of colony-forming units/plate by the dilution and volume factor [36]. 

### Canal disinfection

Root canal samples were divided into four main groups (*n* = 10), and a description of microbiological samples is shown in Table 1:

### Group I

After the incubation with the bacterial suspension, root canals were irrigated with 5 ml of 2% chlorohexidine solution and left for 1 min, followed by the insertion of three sterile paper points using a sterile tweezer to take the second sample [37]. (S2a).

### Group II

After the incubation with the bacterial suspension, root canals were dried and irradiated by a diode laser (Elexxion, Claros Plcco, Germany) with output power 2w for 5s and a wavelength of 810 nm in continuous mode. An optical fiber 200 μm in diameter was inserted into a canal 1 mm shorter than the working length. Four times irradiation repetition at 10-second time interval with an energy density 2.68 J/mm^2^, and then the bacterial counting was performed [38]. (S2b).

### Group III

After the incubation with the bacterial suspension, they were irrigated with 5 ml of Ethanolic extract of propolis solution and left inside the root canal for 1 min then three sterile paper points were inserted by sterile tweezer in the root canals to take the second bacterial sample [19]. 

(S2c)

### Group IV

**Fig. 1:**
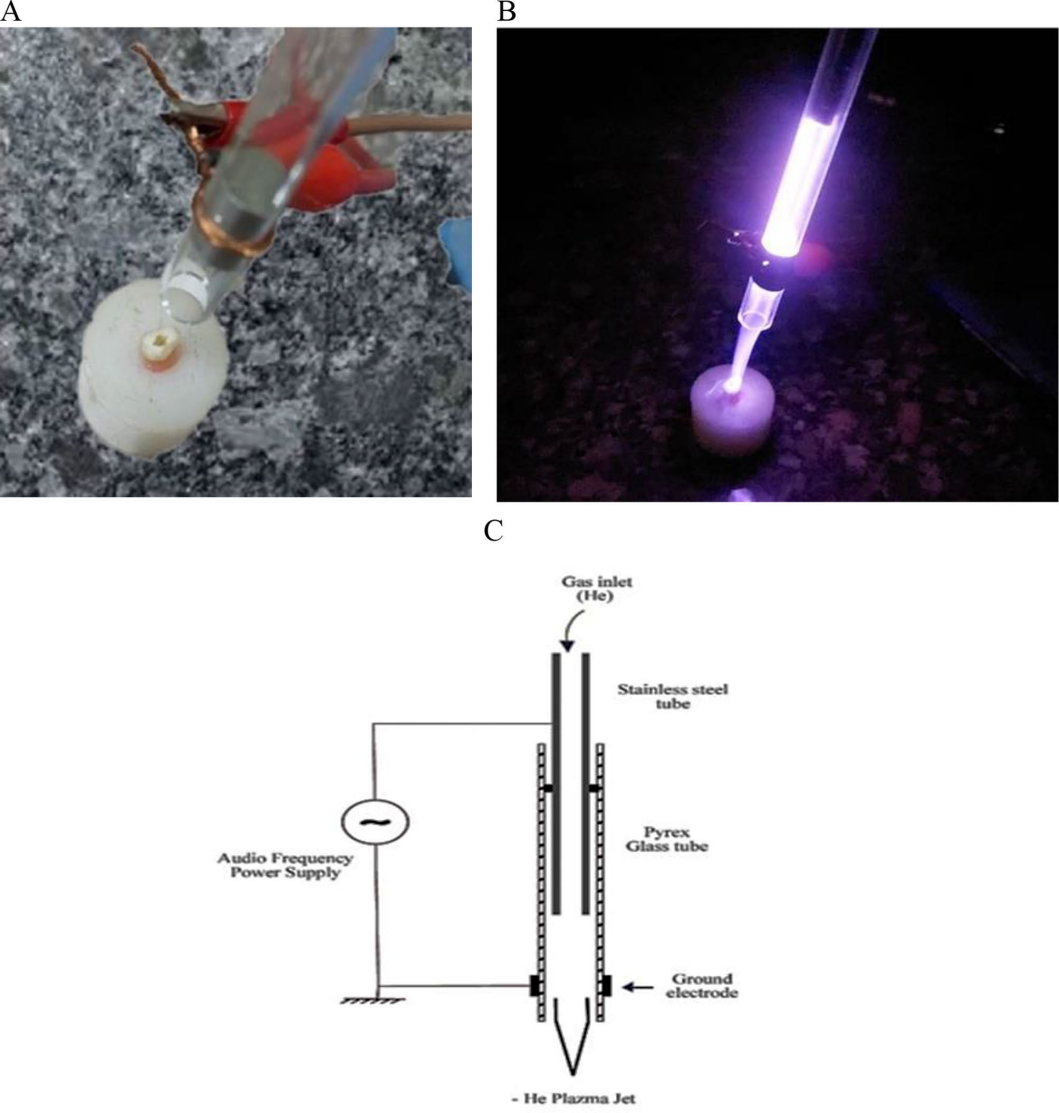
Root canal disinfection with NTPP: (**A**) Primary root canal sample mounted on an acrylic mould; (**B**) Plasma Radiation of the primary root canal sample, (**C**) A schematic diagram of the experimental Helium plasma jet device

fter the incubation with bacterial suspension, root canals were subjected to experimental NTPP (Center of Plasma Technology-Faculty of Science- Al Azhar University-Cairo-Egypt) with ionized helium gas utilizing a cold plasma hand-piece with 16.1 kHz frequency, 3.4 kV input power, 4 L/min flow rate, and rigour of 4 for 1 min (Fig. 1, A-B),. A nozzle tip distance was 4 mm from the specimen exterior surface (Fig. 1, C), and then bacterial counting was performed (S2d) [39]. 

**Table 1:** Description of microbiological samples

Samples grouping	Subgroups	Description of all microbiological samples.
S1		Samples after inoculation of bacterial suspension inside root canals and before disinfection of canals
S2	S2a	Samples after irrigation of root canals with 2% chlorohexidine for 1 min.
	S2b	Samples after treatment with 810 nm diode laser for 5 s.
	S2c	Samples after irrigation of root canals with 5 ml of ethanolic extract of propolis solution for 1 min.
	S2d	Samples after treatment with NTPP for 1 min.

## Results

Data were statistically analyzed utilizing SPSS version 26 by one-way ANOVA for (general comparison) followed by Bonferroni post hoc analysis for pairwise comparisons at 0.05 significance level.

### I-Comparison within groups regarding colony-forming units (log CFUs / mL) before and after irrigation

**Fig. 2:**
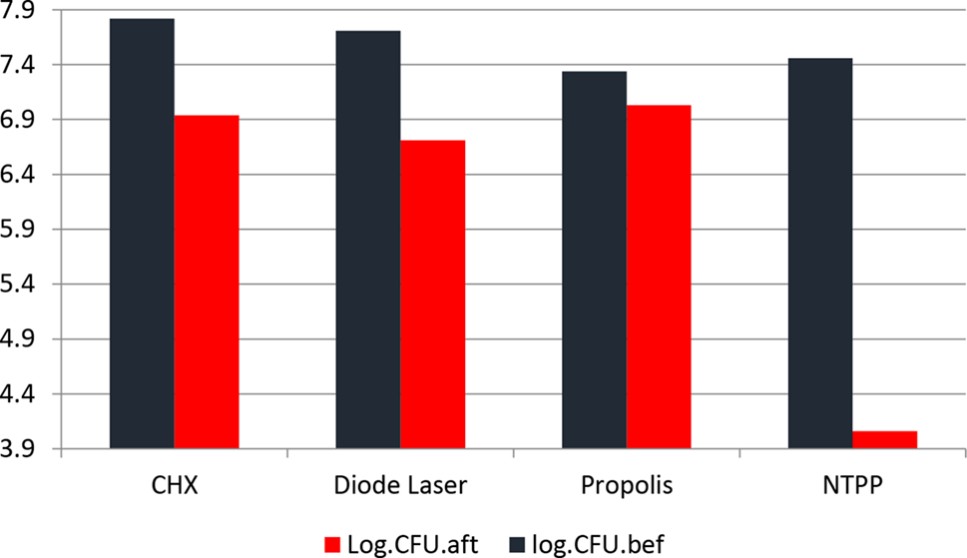
Bar graph showing colony-forming units before and after different disinfecting protocols (log CFUs / ml)

Table 2 and Fig. 2 represent the colony-forming units before and after irrigation (log CFUs/Ml).

There was a significant difference between measured values before and after four irrigation types (*p* < 0.001) for CHX, Diode Laser, NTPP, and (*P* = 0.035) for Propolis. The highest values of colony reduction measured before and after irrigation were for NTPP (4.06 ± 0.88).

**Table 2:** Colony-forming units before and after the irrigation (log CFUs / mL) (*n* = 9)

Type of irrigation	Log CFUs/ml (mean ± SD)		95% confidence interval of difference		
	Before irrigation	After irrigation	Lower	Upper	*P* value
Chlorohexidine	7.82 ± 0.08	6.94 ± 0.27	0.68	1.08	< 0.001*
Diode Laser	7.71 ± 0.16	6.71 ± 0.26	0.86	1.1	< 0.001*
Propolis	7.34 ± 0.03	7.03 ± 0.46	0.03	0.65	= 0.035*
NTPP	7.46 ± 0.04	4.06 ± 0.88	2.23	3.36	< 0.001*

*; significant (*p* ≤ 0.05) ns; non-significant (*p* > 0.05)

### II- Comparison between groups regarding reduction in colony forming units count after the irrigation

**Fig. 3:**
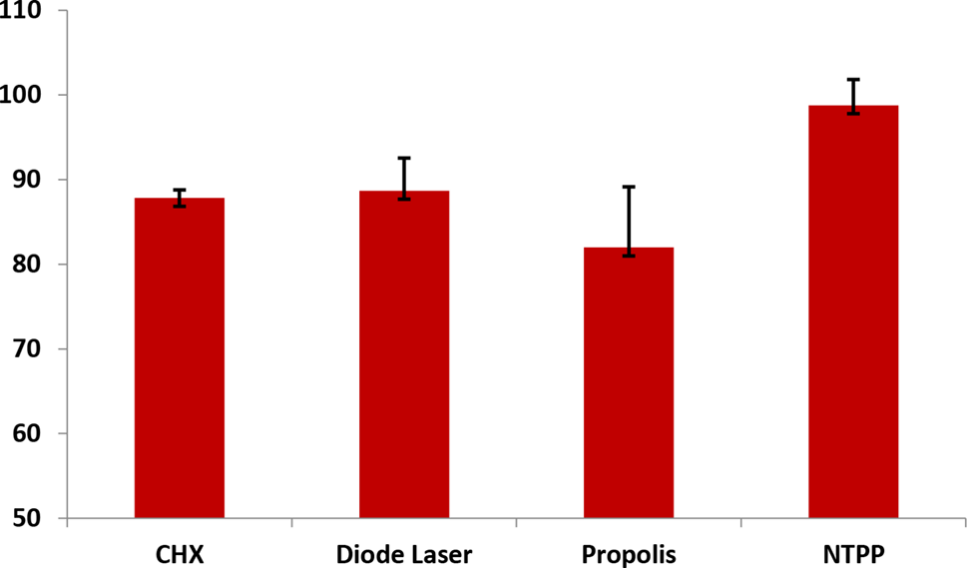
The mean percentage of reduction (%) in colony-forming units in each group after the intervention for each irrigation type

Table 3 and Fig. 3 represent the reduction percentage in colony-forming units in each group after the irrigation compared with the baseline (before the irrigation).

The highest reduction in colony-forming units count was noticed in NTPP group (98.79%), while the least reduction in colony-forming units count was noted in Propolis group (81.99%).

Comparison of the four groups regarding bacterial count reduction by one-way ANOVA displayed a significant variation between groups (*P* < 0.001).

It showed that CHX was significantly more efficacious than Propolis in reducing bacterial count (*P* = 0.001). Diode Laser was significantly more efficacious than Propolis in bacterial count reduction (*P* < 0.001). NTPP was significantly more efficacious than CHX (*P* < 0.001), Diode Laser (*P* < 0.001), and Propolis (*P* < 0.001) for decreasing bacterial count. No other considerable distinctions were recorded (*p* > 0.05).

**Table 3:** Pairwise comparisons of the groups regarding the percentage of reduction in colony count using Post hoc analysis

(I) Group	(J) Group	Mean Difference (I-J)	Std. Error	Sig.	95% Confidence interval	
					Lower Bound	Upper Bound
**CHX**	**Diode Laser**	− 0.847	1.46	1.00	-4.87	3.18
	**Propolis**	5.85^*^	1.46	0.001	1.82	9.88
	**NTPP**	-10.94^*^	1.49	.<001	-15.05	-6.83
**Diode Laser**	**CHX**	0.847	1.46	1.00	-3.18	4.87
	**Propolis**	6.70^*^	1.43	< 001	2.75	10.64
	**NTPP**	-10.09^*^	1.46	< 001	-14.12	-6.067
**Propolis**	**CHX**	-5.85^*^	1.46	0.001	-9.88	-1.82
	**Diode Laser**	-6.70^*^	1.43	< 001	-10.64	-2.75
	**NTPP**	-16.79^*^	1.46	< 001	-20.82	-12.76
**NTPP**	**CHX**	10.94^*^	1.49	< 001	6.83	15.05
	**Diode Laser**	10.09^*^	1.46	< 001	6.06	14.12
	**Propolis**	16.79^*^	1.46	< 001	12.76	20.82

*. The mean difference is significant at the 0.05 level

## Discussion

This study compared the efficacy of NTPP, diode laser, propolis, and CHX for sanitization of deciduous anterior root canals colonized with E. faecalis after sterilization by gamma radiation. Primary Root canal samples were used to simulate the children’s mouth. This agreed with a previous study [40] that compared the antimicrobial effect of different irrigants against E. faecalis in deciduous anterior teeth.

Standardized root canals with 8 mm length to act as reservoirs for bacterial suspension were prepared from primary anterior root canals [41]. 

Gamma ray (25 kGy) was used for sterilization as it completely eradicates all bacterial forms that may be found in the root canals, without detectable changes in dentinal tissues while other methods (dry heat, ethylene oxide, and autoclaving) of sterilization may affect dentin [42]. 

E. faecalis was selected for the study as it is the most dominant species in reinfection cases [43].

A classic colony counting technique [44] was used in this study; to determine the antibacterial efficacy of the tested irrigant by counting the vital E. faecalis colonies on the media plates.

Results proofed that all the used methods; reduced colony count, the highest reduction was noted in NTPP treated group, while the most minor reduction was noticed in Propolis treated group، this agrees with a previous research [45] which reported that NTPP lessened E. faecalis colony count on a glass slab after two minutes. It was concluded that NTPP was more efficient than photodynamic therapy and diode laser for removal of E. faecalis in primary root canals and can act as an alternate to 2.5% NaOCl irrigation [39] and this is in agreement with the current study findings. Considering the significance of rapid proceedings in pedodontics, a 99% reduction in colony-forming units after 1 min of NTPP application was reported [39]. Such an agreeable result in root canal disinfection in a brief period; recommends its clinical application [39].

NTPP generates reactive species, including ions, electrons, and neutral particles, which interact with microbial cells and biofilms present within the root canal system. The oxidative stress induced by these reactive species leads to cellular damage, disrupting the integrity of bacterial membranes and causing cell death. Additionally, NTPP promotes the formation of reactive oxygen and nitrogen species that enhance the antibacterial effect while preserving the dental tissue [46].

Antimicrobial efficacy of NTPP was conditioned with time [47] and 3 min of exposure time recorded superior results, a significant reduction of biofilm microorganisms on helium or He/O_2_ NTPP exposure for 4, 6, and 8 min was also reported [48].

This study used 810 nm diode lasers [49] in accordance with a study which concluded that 810 nm diode laser causes a considerable decrease in E.faecalis colony count, in a continuous mode than pulse mode [50].

Disinfection with 2% CHX dramatically reduced the microbial colony count; this agrees with a previous study [51] that compared the effect of 2% CHX and diode laser on root canal disinfection and concluded that both techniques decreased bacterial count significantly whereas the diode laser was more efficacious than 2% chlorhexidine. The results showed that NTPP was more effective in reduction of bacterial count than CHX 2%. Nevertheless the antibacterial efficiency of the plasma device in another study [52] was analogous to that of 2% CHX.

Propolis was used as a natural alternative irrigant in the current study as it affects the bacterial cell wall, causing structural and functional damage. It produced the most minor reduction in bacterial count. NTPP, CHX, and diode laser were significantly efficient than Propolis for bacterial count reduction, this agrees with a previous study [53] that found that Chlorhexidine was more effective than Propolis and there was enhanced antibacterial capacity with increasing concentrations.

However, the results of this study disagreed with another research [54] that deduced that diode Laser and Brazilian Propolis have equal effect as CHX in cavity disinfection.

The results of this study can be justified by another study [55], which stated that diode Laser antibacterial mechanisms is due to thermal and photo disruptive effects that causes cell wall lethal damage; also it can be attributed to the greater depth of Laser radiation penetration into the dentin, surpassing the effect range of chemical disinfectants.

## Conclusion

Under the current study operating conditions, utilizing non-thermal plasma has a distinct advantage for achieving total inactivation compared with the conventional procedures used in endodontic clinical applications in a short time interval.

## Electronic supplementary material

Below is the link to the electronic supplementary material.

Supplementary Material 1

## Data Availability

The data that support the findings of this study are openly available with the authors.Amira-samy@buc.edu.egShymaashishiny.26@azhar.edu.egyomnaosama.26@azhar.edu.eg.

## Abbreviations

NTPP: Non-thermal pressure plasma
CHX: Chlorhexidine
E. faecalis: Enterococcus Faecalis
NaOCl: Sodium hypochlorite
RONS: Reactive oxygen and nitrogen species
ATCC: American Type of Culture Collection
CFUs: Colony-forming units
