# Supplementary material for: Efficacy of non -thermal pressure plasma versus other modalities for disinfection of primary root canals
Source: BMC Oral Health. 2025 Jan 11;25:54. doi: 10.1186/s12903-024-05349-5 (PMC11725210; doi:10.1186/s12903-024-05349-5)
Supplement: Supplementary file 1 — Supplementary Material 1 [file 12903_2024_5349_MOESM1_ESM.pdf]

RESEARCH ETHICS COMMITTEE
FACULTY OF DENTAL MEDICINE for Girls
AL-AZHAR UNIVERSITY

Principal Investigator: **Dr.** Shaymaa A. El Shishiny, Yomna O.Morad , Rania I. Hindi, Amina M. Motasem, ,Asmaa A. El Sheshiny, Dalia m.alramady, Amira M. Samy

Department: Postdoctoral Researchers

**Ethics Committee Board Decision**
The Research Ethics Committee of the Faculty of Dental Medicine for Girls, Al-Azhar University is constituted and operates according to ICH GCP guidelines and applicable local and Institutional regulations and guidelines which govern IRB operation. The committee met on March 2024.
**Study Title: Efficacy of Non -Thermal Pressure Plasma versus other modalities for disinfection of primary root canals**
**Protocol Code**: **P-PD-24-03**
**Final Code**: **REC-PD-24-03**

Dear Dr.

The Research Ethics Committee, Faculty of Dental Medicine for Girls, Al-Azhar University met and reviewed the presented documents after completing the research which in consensus with the data obtained from the basic study protocol and, this is a final research approval for documentation and publication proposes.

Yours sincerely,

Chairman of Research Ethics Committee

Address: Faculty of Dental Medicine for Girls, Al-Azhar University, Nasr City.
Email: ethics.committee@azhar.edu.eg /
azhardentistry.rec@gmail.com
